# Active and passive chlorophyll fluorescence measurements at canopy level on potato crops. Evidence of similitude of diurnal cycles of apparent fluorescence yields

**DOI:** 10.1007/s11120-022-00995-8

**Published:** 2022-12-17

**Authors:** Hildo Loayza, Ismael Moya, Roberto Quiroz, A. Ounis, Yves Goulas

**Affiliations:** 1 International Potato Center (CIP), Headquarters, P.O. Box 1558, Lima, Peru; 2 LMD/IPSL, CNRS, ENS, Ecole Polytechnique, Sorbonne Université, 91128 Palaiseau, France; 3 CATIE-Centro Agronómico Tropical de Investigación y Enseñanza, Turrialba, Cartago, 30501 Costa Rica

**Keywords:** LED-induced fluorescence, Solar-induced fluorescence, Fluorescence yield, Steady-state fluorescence, Potato

## Abstract

We performed active and passive measurements of diurnal cycles of chlorophyll fluorescence on potato crops at canopy level in outdoors conditions for 26 days. Active measurements of the stationary fluorescence yield (Fs) were performed using Ledflex, a fluorescence micro-LIDAR described in Moya et al. (Photosynth Res 142:1–15, 2019), capable of remote measurements of chlorophyll fluorescence under full sun-light in the wavelength range from 650 to 800 nm. Passive measurements of solar-induced fluorescence (SIF) fluxes were performed with Spectroflex, an instrument based on the method of filling-in in the O_2_A and O_2_B absorption bands at 760 nm (F760) and 687 nm (F687), respectively.

Diurnal cycles of Fs showed significant variations throughout the day, directly attributed to changes in photosystem II yield. Contrasting patterns were observed according to illumination conditions. Under cloudy sky, Fs varied in parallel with photosynthetically active radiation (PAR). By contrast, during clear sky days, the diurnal cycle of Fs showed a “M” shape pattern with a minimum around noon.

F687 and F760 showed different patterns, according to illumination conditions. Under low irradiance associated with cloudy conditions, F687 and F760 followed similar diurnal patterns, in parallel with PAR. Under high irradiance associated with clear sky we observed an increase of the F760/F687 ratio, which we attributed to the contributions in the 760 nm emission of photosystem I fluorescence from deeper layers of the leaves, on one end, and by the decrease of 687 nm emission as a result of red fluorescence re-absorption, on the other end.

We defined an approach to derive a proxy of fluorescence yield (FYSIF) from SIF measurements as a linear combination of F687 and F760 normalized by vegetation radiance, where the coefficients of the linear combination were derived from the spectral transmittance of Ledflex. We demonstrated a close relationship between diurnal cycles of FYSIF and Fs, which outperformed other approaches based on normalization by incident light.

## Introduction

The chlorophyll fluorescence (ChlF) of the vegetation is becoming a principal tool for monitoring the photosynthetic activity at the canopy level. This signal is related to the existing competition for the absorbed light energy between several deactivation pathways within the collecting antenna of photosystem I (PSI) and photosystem II (PSII). They encompass (1) the photosynthetic conversion at the reaction centers, (2) the internal conversion into heat, and (3) the fluorescence emission. Information on photochemistry can be inferred from the variation of the fluorescence emission (Kautsky and Hirsch 1931; Genty et al. 1990).

Chlorophyll fluorescence in natural conditions (outdoor and under full sun-light) varies much less than during an induction after dark adaptation but depending on stress conditions and their variations are readily detectable (Lopez 2015; Moya et al. 2019).

For the last 20 years, passive fluorescence has been preferentially detected using the filling-in of the atmospheric absorption band B (O_2_B) at around 687 and band A (O_2_A) at 760 nm (Moya et al. 1999). Band B is located almost exactly at the first peak of the fluorescence emission, whereas band A is not far from the second fluorescence peak. The interest in the passive method has grown because, although attenuated by the terrestrial atmosphere, the filling-in of these bands was still detectable from a sensor carried by a satellite. However, we voluntarily limited our interest to field measurements.

### Diurnal cycles of passive fluorescence measurements

Significant progress has been made in solar-induced chlorophyll fluorescence (SIF) diurnal cycles at the canopy level and reported flux measurements on different crops. For instance, Louis et al. (2005); Daumard et al. (2012); Goulas et al. (2017); Du et al. (2019), with different instruments, presented diurnal cycles of SIF at O_2_B and O_2_A on pines of the boreal forest, wheat, or maize fields, respectively, showing different shapes along the day. Other works also reported fluorescence flux diurnal cycles within the two bands (Daumard et al. 2010; Xu et al. 2021), but the resolution of the data presented is relatively low.

PSII predominantly produces the fluorescence flux retrieved in the O_2_B band, while the O_2_A band has fluorescence contributions of PSII and PSI (Boardman et al. 1966; Govindjee 1995). Reabsorption plays also an important role. The red fluorescence emission comes principally from the upper layers of the leaf, while the far-red fluorescence is emitted from much deeper layers of the leaf, as compared to red fluorescence. As a result, the fluorescence flux retrieved in the O_2_B band and O_2_A band are sampled in different layers of leaf with different light intensities. In consequence, their kinetics are different.

In addition, recent works with dilute suspensions of green unicellular algae (*Chlorella vulgaris*) using a highly sensible Multi-Color-PAM fluorimeter (Heinz Walz GmbH, Germany) reported evidences a variable fluorescence of PSI (Schreiber and Klughammer 2021). However, in this work, a contribution of variable PSI fluorescence emission was not considered since we worked with green leaves and under natural light conditions that didn’t allow an induction with completely oxidized plastoquinone.

To study the diurnal kinetics of fluorescence retrieved from O_2_ bands, we built a new version of Spectroflex, first described by Fournier et al. (2012). It is a spectrometer-based passive instrument to continuously recover chlorophyll fluorescence emissions within O_2_A and O_2_B bands from potato crops.

We chose the potato crop due to its importance as a food security crop, as indicated by the Food and Agriculture Organization of the United Nations (FAO), due to its widely adaptive range, excellent yield potential, and high nutritional value (Devaux et al. 2014). In addition, to our knowledge, there are only a few studies of SIF measurements on potato crops at the canopy level, except for recent studies performed by Xu et al. (2021).

### Active fluorescence measurement at canopy level

In contrast to SIF measurements, only a limited number of works on light-induced fluorescence (LIF) measurements in outdoor conditions are reported. For example, Rosema et al. (1998) presented diurnal cycles of Fs measured on poplar trees placed inside a growth cabinet with a glass wall. Recently Moya et al. (2019), using Ledflex, a diode-based micro-lidar to measure continuously at a distance (4 m) and canopy level, reported first time Fs diurnal cycles on pea, mint, and grass plants for several days under natural conditions. In addition, they presented a straightforward methodology to detect water stress by comparison of Fs values at darkness and noon.

In this work, we used a Ledflex replica. For 26 days, diurnal LIF cycles were measured on potato crops' foliage over the same target as Spectroflex.

### Diurnal cycle of passive and active measures of ChlF

Combined LIF and SIF measurements at the foliage scale are scarce, and we found just one such study. Louis et al. (2005) measured scot pines with both methods. They presented the first approximation of passive fluorescence measurements using the Passive Multi-wavelength Fluorescence Detector (PMFD), described by Evain et al. (2001), together with the active fluorescence measurements using the micro-LIDAR FIPAM (Flexas et al. 2000). The comparisons showed a discrepancy at solar noon, and the authors attributed it to differences in the structure of the targets measured by both instruments. For example, FIPAM saturates and measures fluorescence at 2 m covering an area of 3 mm × 20 mm. In comparison, PMFD covers a full tree at 40 m.

In this study, we present, for the first time, a comparison of active and passive diurnal cycles of stationary fluorescence at the canopy level. The objectives of this work were the following:

- To retrieve cycles of passive fluorescence from potato canopy O_2_A and O_2_B bands and compare their diurnal variations.

- Using the same target, we perform active fluorescence measurements to gain insights into stationary fluorescence variations.

- Compare active and passive fluorescence yields of potato plants.

## Materials and methods

### Environment and plant material

The field experiment was carried out at the International Potato Center (CIP) experimental station in Lima-Peru (12.08°S, 76.95°W, 244 m.a.s.l.), from July 5th to October 03th, 2017. The study site is a subtropical arid desert climate with cloudy skies during the first hours of the day (autumn–winter), 19.7 °C of average annual temperature, and 6.0 mm of annual total precipitation (2013–2017, CIP Meteorological Station). The potato variety studied was UNICA (CIP code: 392,797.22), an improved genotype with partial salt and high-temperature tolerances, PVY virus resistance, and susceptibility to leafminer fly (*Liriomyza huidobrensis*) (Gutiérrez et al. 2007).

### Chlorophyll content

Leaf chlorophyll content was estimated with a chlorophyll meter SPAD-502 (Minolta, Ramsey, NJ, USA). It is expressed as SPAD units and it was recorded as the mean value of 20–30 measurements per leaf.

### Active instrument: ledflex

Ledflex is a laboratory-designed fluorescence micro-LIDAR for continuous open field measurement of relative stationary fluorescence yields (Fs) from vegetation foliage. Fluorescence is induced by a pulsed artificial light source synchronized with a fast detection system. This type of technique is known as light-induced fluorescence (LIF). For a detailed description of Ledflex, see Moya et al. (2019).

A Ledflex replica was implemented for this work, and its main features include

### The excitation light source

The light source consists of four identical blue light-emitted diodes (LEDs) (LED470L, Thorlabs, Maisons-Laffitte, France). These LEDs emit at a central wavelength of 470 ± 5 nm with a full width at half-maximum (FWHM) of 22 nm, mounted in series and powered under 3 A by a source of 22 V. The light source is controlled by an electronic circuit that adjusts the light intensity and the operation mode of the LEDs to work in pulses with a repetition rate of 10 ms. Although the frequency is low, it is sufficient to follow the variations of natural light, whose transition time is about 1 s. The pulse duration was tuned to 4.5 µs; thus, the mean value of irradiated light from Ledflex avoids generating any change in plant photosynthesis. In addition, the power supply board generates digital outputs to synchronize the acquisition system with the fluorescence pulses induced by the LEDs.

### The fluorescence detection optics

It is based on a 6-inch diameter Fresnel lens (Edmund Optics, France) that focuses the fluorescence light on a 10 mm × 10 mm PIN photodiode (S3590-01, Hamamatsu, France) associated with a combination of a long-pass filter (Schott RG665, Edmund Optics, U.K.) and a short pass filter (*λ* < 800 nm, Edmund Optics, U.K.) that limits the spectral detection range from 650 to 800 nm. Furthermore, a 3 inches focal was chosen to collect a surface of about 55 cm in diameter at a distance of 4 m. Such an area is large enough to provide an excellent spatial integration in the case of potato crops. Finally, a PVC (polyvinyl chloride) tube of 6 inches in diameter was used to enclose both the optic and detection system in a waterproof case.

### The fluorescence acquisition system

Signal conditioning is based on an electronic card at the bottom of the fluorescence detection optics. Its function is to transform the output current of the PIN photodiode into signals directly measurable by the acquisition system. The detected signal at the output of the optical sensor is the sum of a slowly varying signal corresponding to the solar irradiance in the filter bandwidth reflected by vegetation and a fast varying signal corresponding to the LEDs-induced fluorescence. After voltage conversion by a trans-impedance amplifier, the two signals are electronically separated by an arrangement of two operational amplifiers with different cut-off frequencies. A fast sample and hold circuit, synchronized with the LEDs’ light pulses, maintains the LEDs’ peak value-induced-fluorescence during analog to digital conversion. A data acquisition device (34970A, Keysight Technologies, Santa Rosa, CA, USA) is used to convert signals to digital numbers either synchronously with a trigger signal to detect Fs or asynchronously for the acquisition of vegetation radiance reflected in the range from 650 to 800 nm, ambient temperature (thermistor RS151–237, Radiospares, France) and incident photosynthetically active radiation (PAR) (radiometer, JYP 1000, Sdec, France). The data was recorded on a portable computer with an average sampling frequency of 1.8 s.

### The signal-to-noise ratio (SNR) of LIF measurements

Tests were performed to measure the ratio between the Fs signal recovered from different targets and the noise. Under open field conditions at approximately solar noon and with Ledflex located about 4 m away and pointing nadir to the canopy of peas plants whose leaves presented SPAD values of 40 (on average), an SNR of 133 was reached. (Moya et al. 2019). Therefore, the SNR value obtained was considered acceptable for the objectives of this study.

### Spectral transmittance of the detection optics

A laboratory test was performed to measure the light transmitted through the Ledflex optics, including the Fresnel lens and the filters, and finally reaching the photodiode. The optics transmittance was measured with a cosine corrector (Ocean Insight, Orlando, FL, USA) adapted to a VIS–NIR spectrometer (H.R. 2000 + , Ocean Insight, Orlando, FL, USA). The corrector was located at the focal point–corresponding to the position of the PIN photodiode–and using a 600 W white light tungsten lamp as a source.

**Fig. 1:**
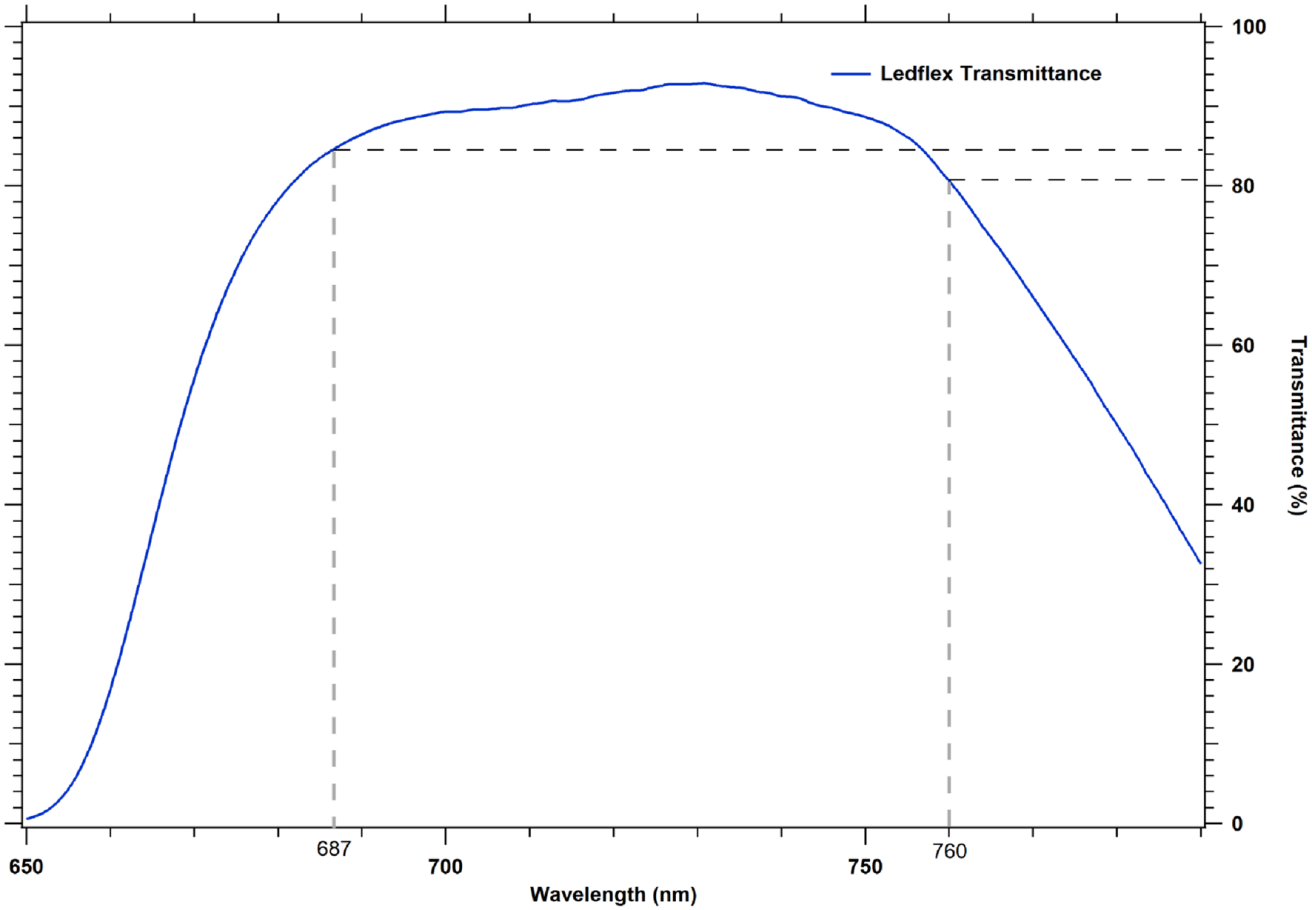
Transmittance of the Ledflex detection optics (blue line). The gray dash lines indicate the positions and intensities corresponding to 687 and 760 nm wavelengths

Figure 1 shows the transmittance spectrum of the Ledflex optics and highlights the contributions corresponding to the wavelengths 760 and 687 nm, giving a transmittance ratio of 0.95.

### Passive instrument: spectroflex

Spectroflex is a laboratory-made instrument designed to accurately and continuously measure the radiance of the vegetation at a high spectral resolution compared to a reference panel under outdoor conditions. With this information, we recovered the solar-induced ChlF (SIF) fluxes at the foliage level using the filling-in method in the A and B absorption bands of atmospheric oxygen (Moya et al. 1999, 2004; Moya and Cerovic 2004) .

The first version of Spectroflex was presented and described by Fournier et al. (2012), and inspired by this work, a new version of Spectroflex was implemented and adapted for this study. A DC rotary solenoid (Magnet-Schultz, Old Woking Surrey, UK) alternated the radiance measurements on the reference panel and vegetation. At the same time, a data acquisition (DAQ) board (NI-USB 6210, National instrument, Texas USA) allowed the incorporation of the PAR sensor (JYP 1000, Sdec, France) readings at each loop of the Spectroflex measurements.

**Fig. 2:**
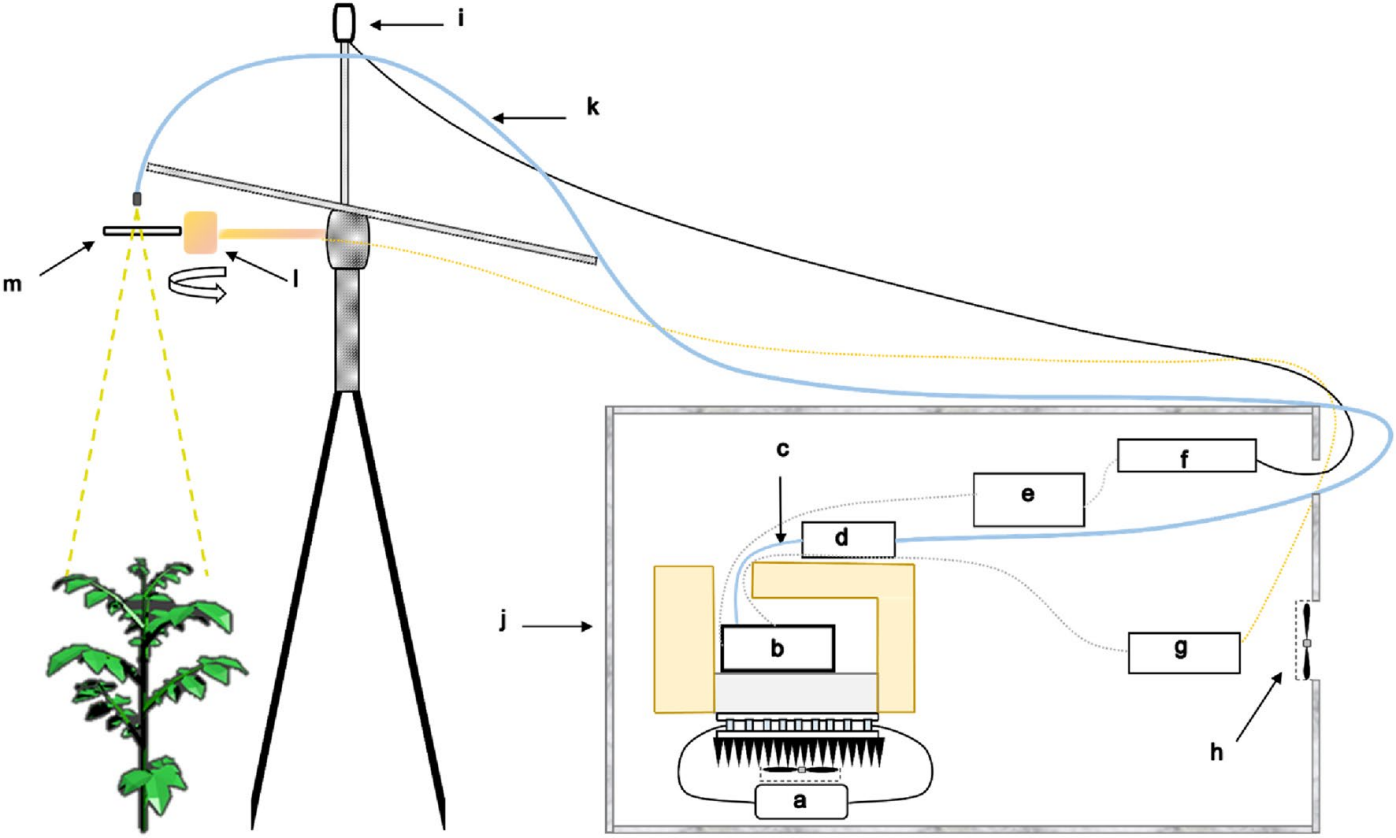
Spectroflex instrument. Electronic devices are placed inside an insulated box to protect them from weather conditions. The different optical and electronic accessories of Spectroflex are: **a** temperature controller, **b** USB 2000 + spectrometer, **c** optical fiber of 13 cm, **d** electronic shutter, **e** minicomputer, **f** Data Acquisition (DAQ) card, **g** power supply for both solenoid and fan, **h** fan, **i** PAR sensor, **j** waterproof, **k** optical fiber of 5 m and collimating lens, **l** rotatory solenoid, stroke up to 95º, and **m** reference panel (PVC)

Figure 2 shows a scheme of Spectroflex installed in open field conditions. The light is transmitted through an optical fiber, placed and fixed on a tripod, with one of its ends pointing in the nadir direction. In the rest condition, it collects the radiance of a reference panel installed on the rotary solenoid, which is managed by the Spectroflex program, which in turn changes the target in a synchronized way. The essential accessories of Spectroflex are described in Fig. 2.

### The spectrometer

A USB2000 + user-configured miniature spectrometer (Ocean Insight, Orlando, FL, USA) was used. A lightweight spectrometer of 190 g was selected since the ultimate goal is to collect passive measurements from an unmanned aerial vehicle (UAV). The spectrometer optic was defined to optimize the radiance measurements in the spectral region around the A-band of atmospheric oxygen absorption. These measurements are achievable with a 1200 lines grating blazed at 750 nm, a focusing lens, a 25-µm entrance slit, a long-pass filter (OF1-GG475), and 16 bits analog to digital (A/D) converter. Furthermore, the detector is a 2048-pixel CCD (Sony ILX511 linear silicon) with a spectral sampling interval of 0.19 nm, resulting in an FWHM of 0.63 nm. Measuring vegetation indexes such as the Photochemical Reflectance Index (PRI) were planned from the onset; therefore, the spectral range was set from 510 to 818 nm.

In addition, the USB 2000 + features a high-speed FPGA controller programmable onboard, a 22-pin connector, and 8-user programmable digital I/Os, also known as General Purpose Input Output (GPIO). It provided the necessary resources to synchronize data acquisition from the spectrometer with the movements of the reference panel controlled by a rotary solenoid (see Fig. 2) and manage the passage of light through an electronic shutter (INLINE-TTL-S model, Ocean Insight, Orlando, FL, USA) both configured in external trigger mode.

To focus and define the field of view (FOV) integrated by the spectrometer, a lens (# 64–772 Edmund Optics, Barrington, NJ, USA), 0.4 numerical aperture, and 6.24 mm effective focal length at 780 nm was used. This setting defines a FOV of 17.5 cm in diameter at a distance of 1 m, verified experimentally.

A relative radiometric calibration was applied to the setup using a calibrated light source LI 1800–02 (LICOR, Lincon, NE, USA). In addition, a wavelengths calibration was performed with a Hg (Ar) calibration lamp (6035 model, ORIEL Instrumentation, Irvine, CA, U.S.) with the support of the NIST Atomic Spectra Database Lines Form.

### Dark measurements

The dark measurements ranged from 2 to 10% of the spectrometer counts, depending on the spectral region and integration time. Therefore, each radiance measurement–whether from vegetation or reference–was accompanied by a dark measurement with the same integration time.

### Reference measurements

A white-roughened PVC board was used as a reference surface to acquire the high-resolution spectrum of incident light. Its reflectance spectrum was previously measured in the laboratory using an ISS-REF model integrating sphere (Ocean Insight, Orlando, FL, USA), obtaining a value of 0.96 ± 0.1% in the VIS/NIR range. The board was then installed on the rotary solenoid and configured to rotate by 90º upon receiving a TTL signal managed by the spectrometer’s GPIO.

A close linear relationship was observed between the radiance of the reference panel at 584 nm (${L}_{ref}\left(584\right)$) which corresponds to the maximum of the reference panel radiance and the readings of the PAR sensor. Hence, after cross-calibration with the PAR sensor, we used ${L}_{ref}\left(584\right)$ as an indicator of the irradiance level, in tight synchronization with the high-resolution spectral information.

### Temperature regulation of spectrometer

The spectrometer was placed inside a tightly closed box made of expanded polystyrene. In order to facilitate heat dissipation, it was fixed on a pile of elements that successively comprised a metal base, a Peltier module (CP1.0–127-05L-RTV, Laird Thermal System, North Carolina, US), a radiator, and finally, a fan to accelerate the cooling of the entire assembly.

A high stability temperature controller (TEC 2000, Wavelength Electronics, Bozeman, Montana, US) collected in real-time the temperature data of a thermistor (TCS610, Wavelength Electronics, Bozeman, Montana, US) of 10 KΩ NTC installed in direct contact between the surface of the conductive base and the spectrometer. The temperature regulation of SpectroFlex was tuned for optimal performance of radiance measurements, reaching a diurnal temperature drift of 0.18 °C on sunny days.

### Spectroflex software

Spectroflex is controlled by a custom program developed in C +  + language, which was implemented in the free Integrated Development Environment (IDE) CodeBlocks 16.01 on a miniature fanless P.C. (Fitlet iA10, Compulab, Yokneam, Israel).

The Spectroflex program allowed the system to record daily cycles of the vegetation’s radiance and reference panel's radiance and their respective dark currents, accompanied by a measure of the incident PAR. The measurements dynamic range was established and adjusted to reach 85% of the maximum number of counts measured over a reference spectral region corresponding to the reference panel and for vegetation, thus improving the SNR. These spectral regions were defined experimentally in tests performed outdoors under natural light conditions.

The exposure times were estimated considering a linear relationship between the measured light and the number of counts obtained on the respective reference spectral region. Moreover, the number of spectra acquisitions was calculated considering a total of two seconds for each measurement cycle. Finally, Spectroflex worked continuously and autonomously with a period of 12 s per loop from 07:00 to 18:00 h.

### Fluorescence retrieval method

The method used to retrieve fluorescence fluxes from radiance spectra was described by Daumard et al. (2010). It is based on the filling-in method of atmospheric oxygen bands A and B proposed by Moya et al. (1999, 2004).

The fluorescence flux corresponding to the atmospheric oxygen absorption band A (O_2_A) was recovered at the bottom of the band at 760.41 nm and hereafter called F760. At the same time, the flux corresponding to the absorption band B of atmospheric oxygen (O_2_B) was recovered at 686.97 nm and referred to as F687 (see Table 1). The following three assumptions were considered:

**Table 1:** Spectral channels used to retrieve fluorescence fluxes in the O_2_B and O_2_A absorption bands and associated form factors of the fluorescence shape

Channels	Central wavelength (nm)	Wavelengths range (nm)	Form factors (K_i_)
O_2_B outband	${\lambda }_{1}$= 683.08	682.77–683.34	0.93
O_2_B outband	${\lambda }_{2}$= 684.95	684.64–685.26	0.99
O_2_B inband	${\lambda }_{3}$= 686.97	686.81–687.12	1
O_2_B outband	${\lambda }_{4}$= 696.97	696.66–697.28	0.90
O_2_A outband	${\lambda }_{5}$= 757.38	756.97–757.80	1.13
O_2_A inband	${\lambda }_{6}$= 760.41	760.14–760.55	1
O_2_A outband	${\lambda }_{7}$= 771.16	770.76–771.57	0.60

- (a) the shape of fluorescence emission spectrum at the canopy level in the vicinity of the O_2_A and O_2_B bands is assumed to be the same as the shape of emission spectrum at the leaf level as discussed by Fournier et al. (2012). The form factors (K_i_) were determined experimentally using a leaf spectrum and are listed in Table 1,

- (b) the reflectance of vegetation in the vicinity of the O_2_A band is assumed to vary linearly with wavelength,

- (c) the reflectance of vegetation in the vicinity of the O_2_B band is assumed to vary in a quadratic way.

F760 can be retrieved by computing the vegetation radiance in the vicinity of the O_2_A band using three channels, two out-band and one in-band, and solving a system of three equations. On the other hand, four channels are used for the O_2_B band, three out-band and one in-band, to retrieve F687 by solving four equations. Selected channels to retrieve F687 and F760 and their respective form factors, *K*_i,_ are presented in Table 1. More details can be found in Daumard et al. (2010); Fournier et al. (2012).

**Fig. 3:**
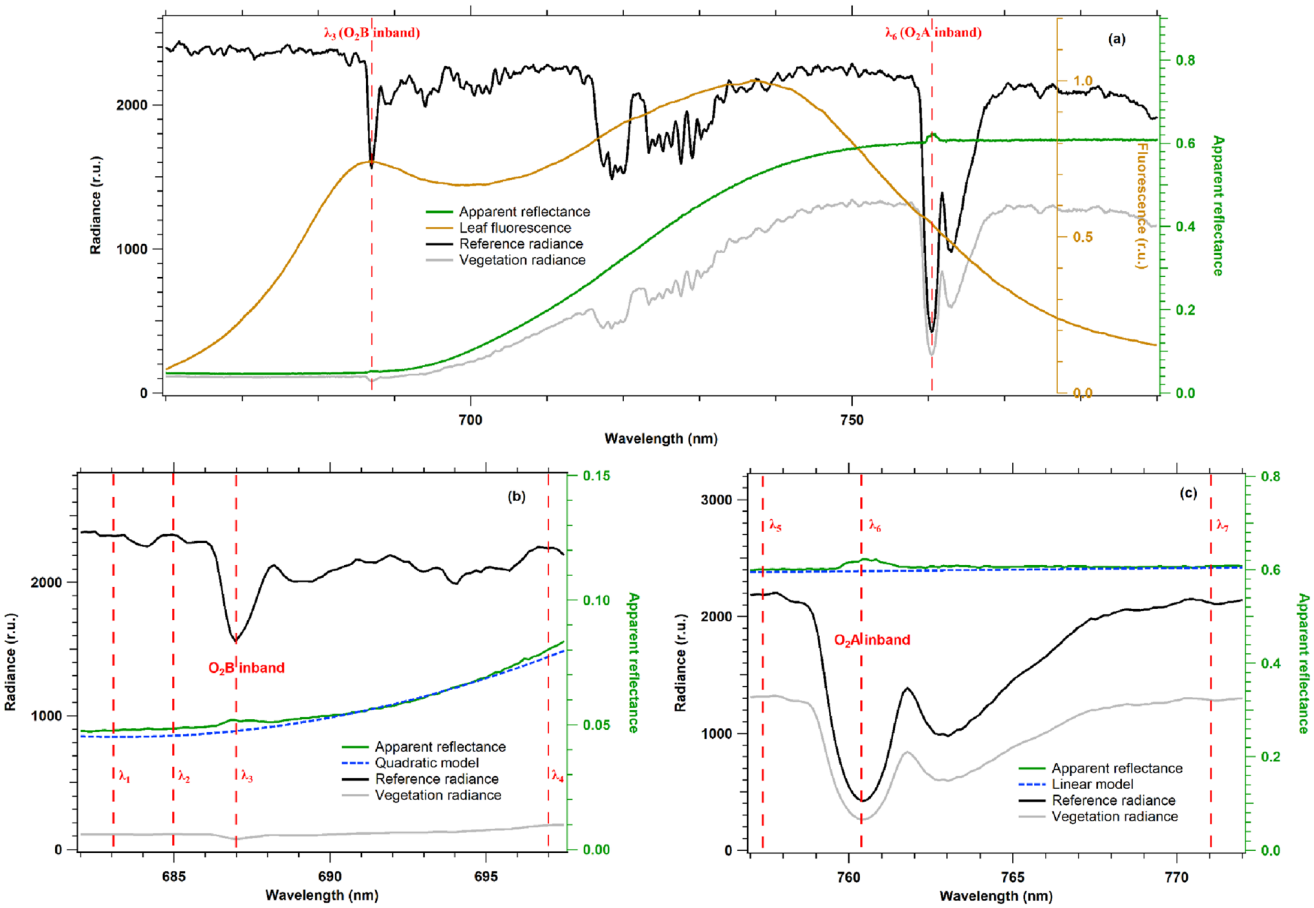
**a** Vegetation and reference panel radiances acquired by Spectroflex (gray and black, respectively). The figure also shows the apparent reflectance at the canopy level (green) and a fluorescence spectrum at the leaf level (SPAD value 31.5) acquired under full sun-light with a fluorimeter already described in Moya et al. (2006) (brown). The position of the O_2_B and O_2_A bands are emphasized (red dotted lines), showing the oxygen absorption features and the small peak in the apparent reflectance induced by the fluorescence filling-in. (**b**) and (**c**) Zooms in the vicinity of O_2_B and O_2_A bands, respectively. The position of the channels used for fluorescence retrieval in each oxygen band is indicated (red dotted lines), the apparent reflectance (green), and the retrieved true reflectance (blue)

An example of data from a measurement cycle of Spectroflex and the corresponding retrieval results are shown in Fig. 3.

### Experimental design and setup to measure fluorescence

The experimental plot had an area of 5 m^2^, 05 furrows containing ten plants per furrow. The furrow’s direction was 16º N, and plants were watered with drip irrigation. Aiming to have a green cover capable of avoiding border effects in active and passive fluorescence measures, the distance among furrows and plants was established at 0.2 m and 0.5 m, respectively.

**Fig. 4:**
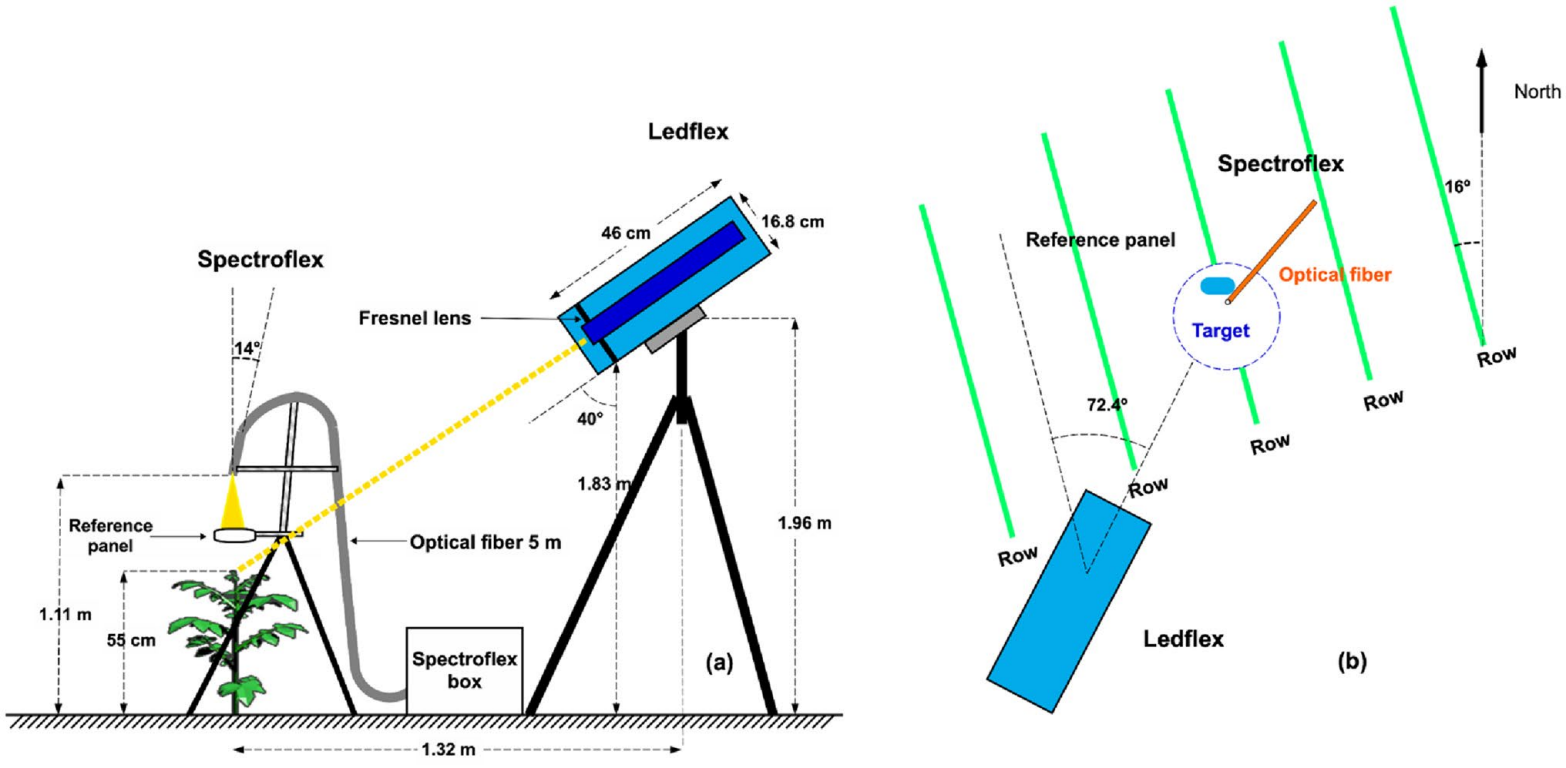
Schematic representation of the geometric configuration of Spectroflex and Ledflex measuring devices as conducted on the plot: **a** Front view and **b** top view. Fluorescence measurements–passive and active–started once the plants reached an average of 55 cm height (maximum vegetation cover)

Fluorescence measurements started once the plants reached their maximum canopy cover. The effective period of fluorescence measurements at canopy level was between 48 and 82 days after planting (DAP), which corresponded from August 22nd to September 25th, 2017, and where the maximum solar height varied from 66.38 to 76.85º. A schematic representation of the configuration of both instruments located in the field is shown in Fig. 4

**Fig. 5:**
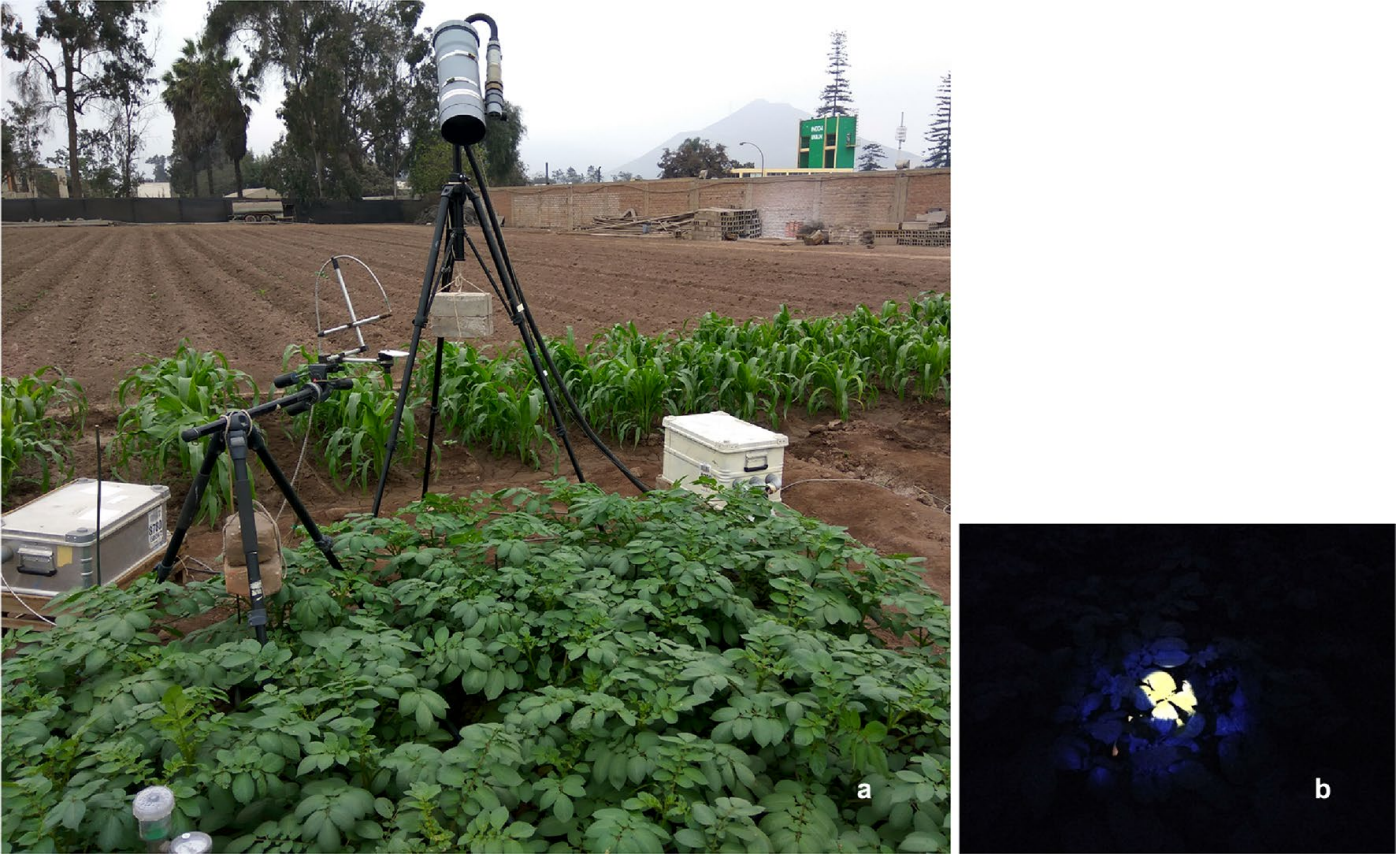
**a** Measurement configuration of Ledflex and Spectroflex over the vegetation cover of a potato plot. **b** Photograph of the plot acquired at night showing the effective area measured by both instruments. The blue color corresponds to the Ledflex FOV (16 cm of radius) and in its interior–in yellow–the Spectroflex FOV (7 cm of radius)

The Spectroflex and Ledflex FOV were adjusted to point and measure on the same target. The calibrations were carried out at night to take advantage of the spot generated on the green cover by Ledflex’s blue LED source. To pinpoint and define the Spectroflex FOV, the end of the primary optical fiber was unplugged and coupled to a tungsten halogen light source (H.L. 2000-FHSA-LL model, Ocean Insight, Orlando, FL, USES). The Spectroflex FOV was contained and aligned to the center of the Ledflex FOV, as shown in Fig. 5b.

### Estimation of fluorescence yields with passive methods

Spectroflex accurately measures the radiances on the reference panel and the vegetation cover, and with this information, it recovers the time series of the fluorescence fluxes in O_2_A and O_2_B bands in relative units. However, the photosynthetic activity is commonly related to ChlF through fluorescence yields, defined as the ratio of the number of photons emitted by fluorescence to the number of photons absorbed by photosynthetic pigments.

The accurate computation of changes in the light absorbed by vegetation under natural illumination during the day in the FOV of the instrument is a challenge. One was to consider the complex interaction between canopy structure and incident light. In addition, re-absorption of fluorescence in the radiative transfer from the leaf surface to the sensor could also play a role. However, we can approximate SIF yields by computing an apparent spectral fluorescence yield (ASFY) as:

1 $$ASFY687=\frac{F687}{APAR}$$

2 $$ASFY760=\frac{F760}{APAR}$$

where: APAR is the PAR absorbed by the vegetation, which varies during diurnal cycles.

In this work, we tested different approximations for APAR to compute diurnal cycles of ASFYs. The results were compared with time series of relative fluorescence yields measured by Ledflex.

Two approximations of APAR were used: (1) Incident PAR, assuming that, given the high fractional vegetation cover of the crop, most of the incident light is absorbed by photosynthetic pigments (2) Radiance of vegetation integrated into a spectral range similar to PAR. The latter approach is motivated by the assumption that, at constant pigment content and leaf position, a close positive relationship is expected between absorbed and reflected light at each surface element of the canopy. We used the data acquired by the Spectroflex's spectrometer to compute: (1) a proxy of the PAR obtained by cross-calibration of the radiance of the reference panel at 584 nm (maximum radiance of panel reference,${L}_{ref}\left(584\right)$ against the PAR sensor, 2) the integrated vegetation radiation. Since the spectral range of the spectrometer is limited to the range 510–818 nm, the spectral portion of the PAR that can be measured covers results from integrating from 510 to 700 nm. Hence, two apparent fluorescence yields per emission channel can be defined and tested for SIF normalization, which are:

3 $$FY687\text{\_}PAR=\frac{F687}{PAR}\sim \frac{F687}{{L}_{ref}\left(584\right)}$$

4 $$FY687\text{\_}Veg=\frac{F687}{{\sum }_{{\lambda }_{i}=510}^{700}{L}_{veg}\left({\lambda }_{i}\right)}$$

5 $$FY760\text{\_}PAR=\frac{F760}{PAR}\sim \frac{F760}{{L}_{ref}\left(584\right)}$$

6 $$FY760\text{\_}Ref=\frac{F760}{{\sum }_{{\lambda }_{i}=510}^{700}{L}_{veg}\left({\lambda }_{i}\right)}$$

where: ${L}_{ref}\left({\lambda }_{i}\right)$ and ${L}_{veg}\left({\lambda }_{i}\right)$ are the radiance at ${\lambda }_{i}$ of the reference panel and vegetation, respectively.

### Assessments of performance fluorescence retrieval

The performance of Spectroflex and the fluorescence flux recovered by Daumard et al. (2010) model were subjected to preliminary open field tests. A material with a known and constant fluorescence emission was used instead of vegetation as the target. It allowed–by comparison–to determine the performance of the set (hardware and software). The material chosen was a white PVC panel whose fluorescence emission was zero.

**Fig. 6:**
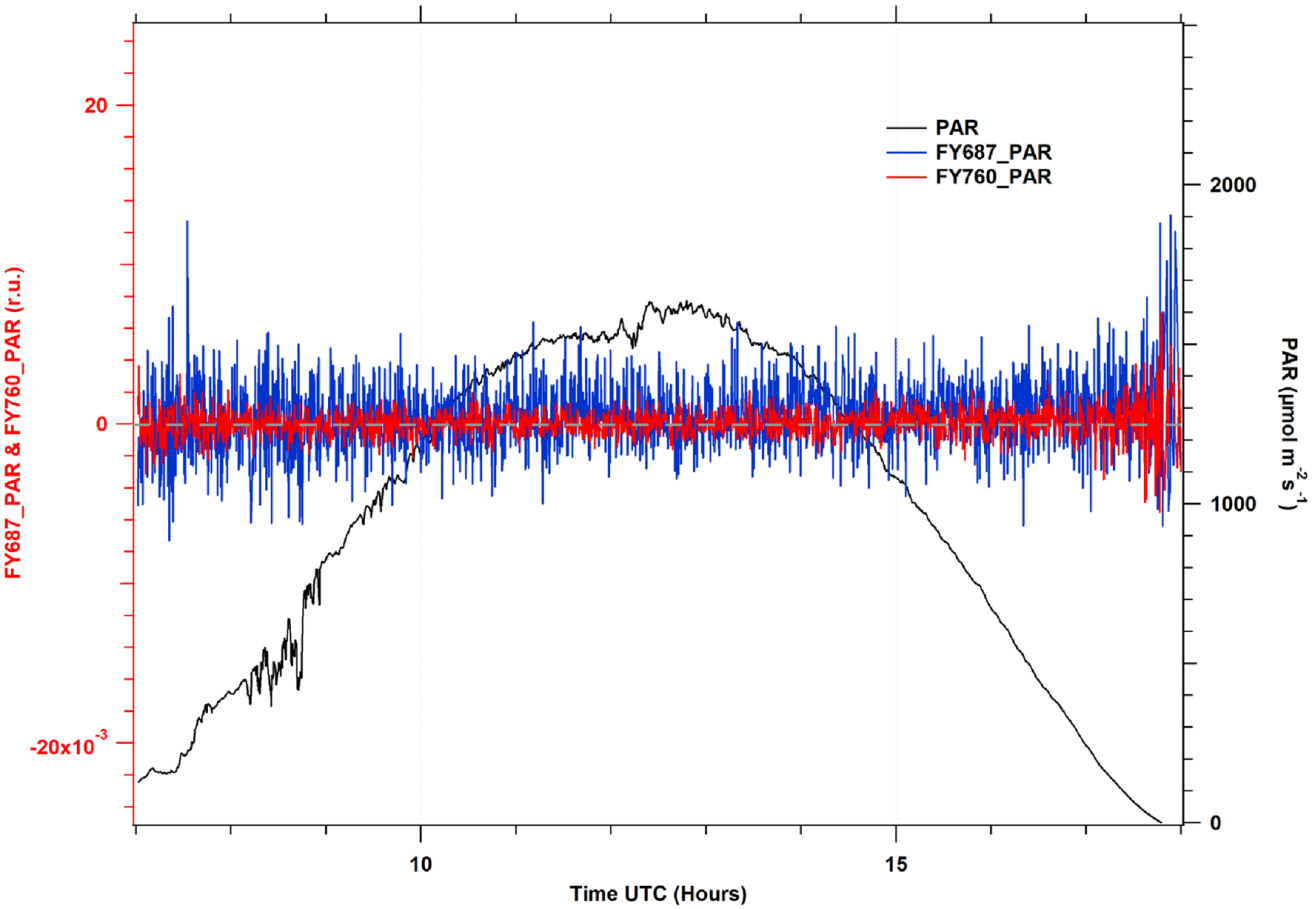
Diurnal cycle of SIF measurements recovered from a white PVC panel. Where: FY760_PAR (red line) refers to ASFY in O_2_A, FY687_PAR (blue line) corresponds to the ASFY in O_2_B and r.u. (relative units)

As it is shown in Fig. 6 the ASFY estimated with incident PAR in O_2_A and O_2_B and measured on a white PVC panel during whole day had a mean value of almost zero.

## Results

### Active fluorescence measurements

**Fig. 7:**
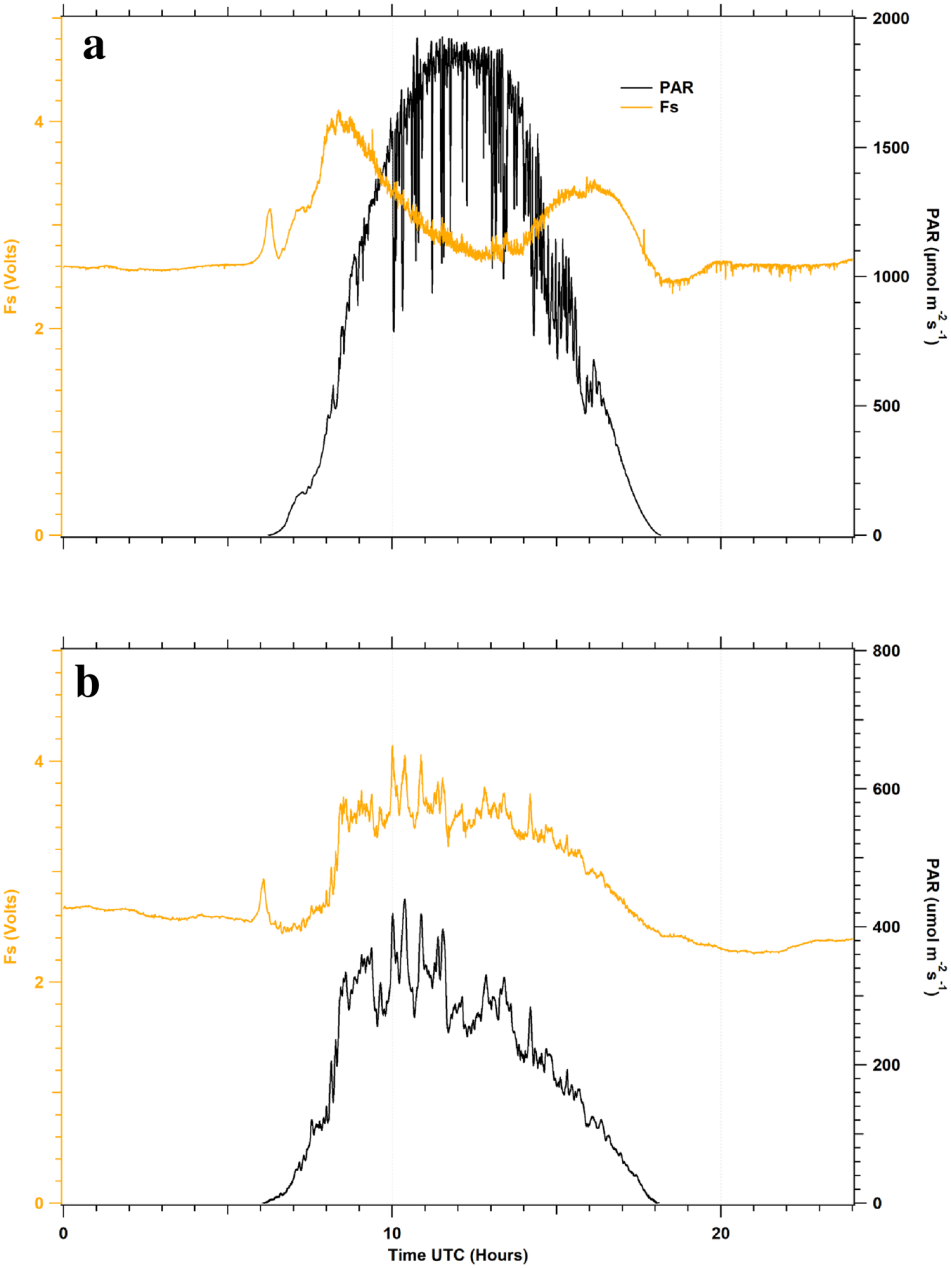
Diurnal cycles of stationary fluorescence yields (Fs) measured over potato canopy under sunny conditions at 56 DAP (**a**) and under cloudy conditions at 71 DAP (**b**). Fs time series changed according to changes in incident PAR

Diurnal cycles of relative fluorescence yield at canopy level over potato plants were measured throughout a campaign (26 days) with Ledflex. These time series were classified according to the sun-light conditions between sunny and cloudy days to show the relationship between Fs changes and incident PAR. One example of each category is shown in Fig. 7.

The cycles (Fig. 7) show that at pre-dawn, i.e., in complete darkness, Fs is relatively constant, at a fluorescence level that can be associated with the F_0_ level (the minimum fluorescence value in the dark when all PSII reaction centers are open, see Van and Snel 1990). Nonetheless, with the first sun-light striking the potato’s foliage, a small and reproducible peak appeared in all recorded cycles, as in Moya et al. (2019). Furthermore, according to PAR intensity, the Fs signal showed different patterns and responses to the incident light.

Under sunny conditions (Fig. 7a), the Fs signal followed the natural changes of PAR until it reached the first maximum value when PAR reached around 600 µmol photons m^−2^ s^−1^. Then it decreased until it reached a minimum local value, whereas incident PAR continued to increase up to the solar noon (maximum intensity). Once this Fs minimum was reached, the signal increased while PAR decreased. When the PAR intensity lowered to around 600 µmol photons m^−2^ s^−1^, Fs finally reached a second local maximum to decrease and followed natural illumination changes. The three extrema of the Fs diurnal cycle generated an M-shape that characterizes the Fs series under sunny conditions, with the second maximum in the afternoon being of lower amplitude compared to the morning one. The experiment obtained similar results for Fs cycles on all sunny days.

Under cloudy conditions (Fig. 7b), the Fs signal closely followed the incident PAR's natural changes over the day. Fs permanently changed in correlation with incident PAR whenever PAR intensity was less than 300 µmol photons m^−2^ s^−1^.

### Passive fluorescence measurements in O_2_A and O_2_B bands

**Fig. 8:**
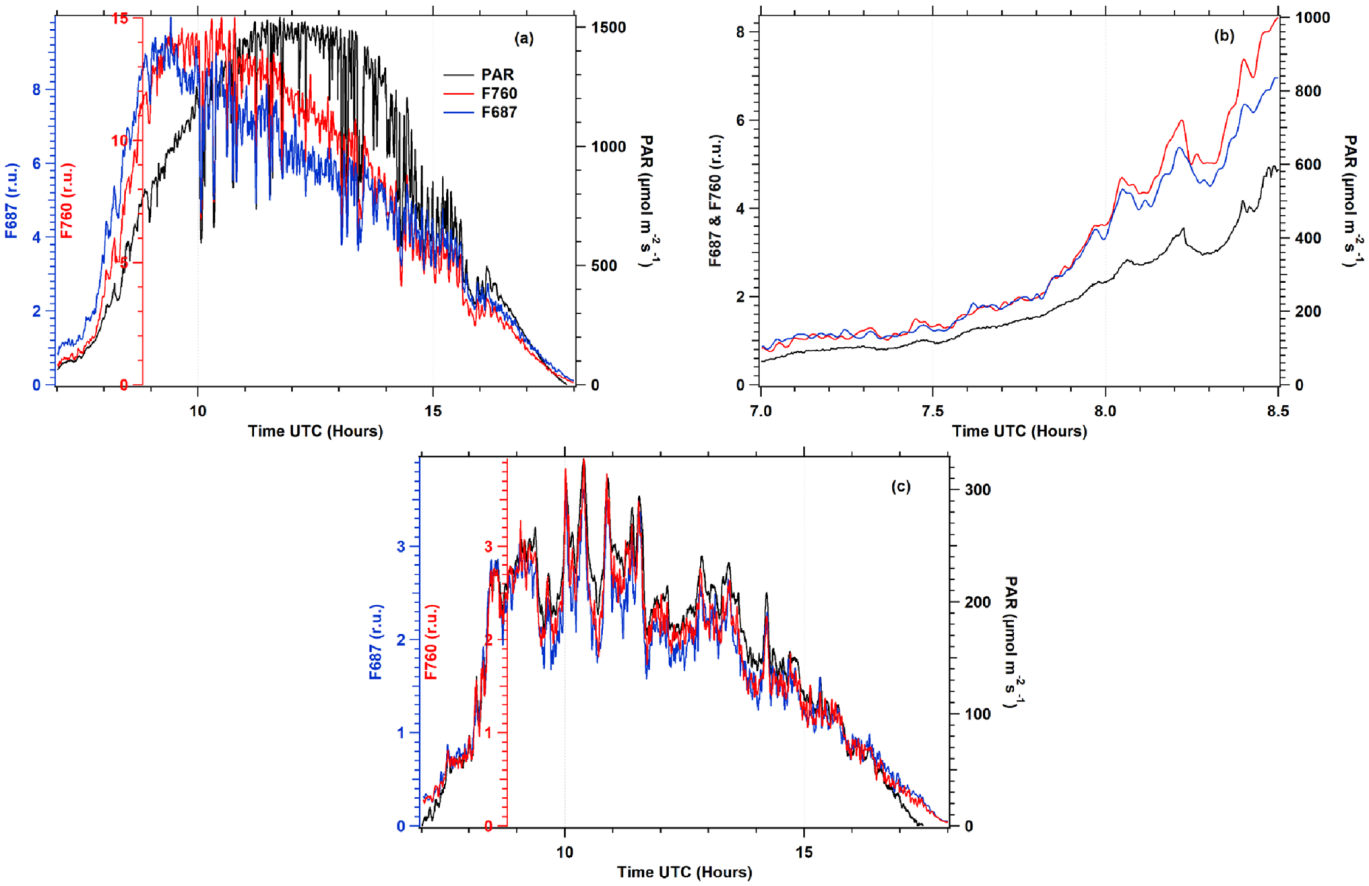
Diurnal cycles of fluorescence fluxes F687 (blue line) and F760 (red line), under sunny day conditions (**a**, **b**) at 56 DAP and a cloudy day (**c**) at 71 DAP. Following the pattern of direct light, the fluorescence fluxes differed in amplitude and shape, whereas under cloudy conditions, both fluorescence fluxes closely followed changes in PAR

During the campaign, time series of fluorescence fluxes from the vegetation cover of potato plants were recovered from two independent channels using the filling-in method of atmospheric oxygen absorption bands at A and B described in the Materials and Methods section. In addition, these diurnal cycles (see Fig. 8) were retrieved using Spectroflex with the method proposed by Daumard et al. (2010).

Figure 8a shows a diurnal cycle example of fluorescence fluxes retrieved under clear days, plotted with independent axes to show their variations with respect to incident PAR. Both diurnal cycles showed similar patterns that changed due to intensity changes of incident light. These diurnal variations were observed in all recorded diurnal cycles. Although similar, F687 and F760 were not identical. During the morning, both time series continued to increase with the increase of PAR until about 1000 µmol photons m^−2^ s^−1^. Nevertheless, after reaching this PAR level, the fluxes continuously decreased while PAR intensity increased. Between 10 and 14 h, F687 decreased faster than F760, and the aspect ratio (F760/F687) was roughly 1.5, whereas it was near one at the beginning of the day (Fig. 8b).

Under cloudy conditions and during the sunrise and sunset of sunny days with PAR intensity less than about 400 µmol photons m^−2^ s^−1^ (see Fig. 8b, c), the diurnal cycles of fluorescence fluxes in red and far-red were very similar in shape and amplitude and closely followed the changes of incident PAR intensity.

On the other hand, considering the whole diurnal cycles measured in this campaign (not shown), we observed that ASFY in 687 nm showed the most significant variations, which may better characterize the PSII electron transfer rate.

## Discussion

### Active measurements of fluorescence cycles

Diurnal cycles of relative fluorescence yields (Fs) were acquired using the active fluorimeter Ledflex. Among several factors that may influence Fs are chloroplast movements as they change leaf absorption and consequently leaf fluorescence (Brugnoli and Björkman 1992; Wada 2013). However, these effects were of small amplitude and occurred at low light (Brugnoli and Björkman 1992). So it was neglected in the following. Measurements were done on a well-watered potato crop and under high light conditions (Fig. 7), similar to those already published using active instruments. See, for example, the works of Rosema et al. (1998), Flexas et al. (2000), and Evain et al. (2004). The comparison with the data presented in Moya et al. (2019) is vital as the micro-LIDAR used in both studies are identical.

As in Moya et al. (2019), Fs reached a maximum at about 8:30 am when PAR exceeded 600 µmol photons m^−2^ s^−1^ (see Fig. 7a), then decreased as Non-Photochemical Quenching (NPQ) developed. NPQ is the regulatory mechanism by which the plants can cope with the excess incident radiation when it exceeds the capabilities of the electron transfer chain (Müller et al. 2001). It involves conformational changes within the light-harvesting proteins of PSII that causes the formation of energy traps. The conformational changes are stimulated by combining a transmembrane proton gradient, PsbS protein, and the carotenoid violaxanthin conversion to zeaxanthin. When PAR decreased in the afternoon, Fs increased due to the relaxation of NPQ (Horton and Ruban 2005).

Further decrease of PAR induced a concomitant decrease of Fs until a level close to F_0_. The M-shape of the diurnal cycle is not symmetrical: the second maximum observed in the afternoon is lower than the first one observed in the morning. We suggest that part of NPQ formed before noon does not fully reverse during the afternoon and needs the entire night to dissipate and start again a new cycle the day after. Figure 7b shows the situation during an overcast day where the PAR stays under 300–400 µmol photons m^−2^ s^−1^. In this case, Fs always closely follow PAR changes. In short, our results agree with the few LIDAR data available in the literature.

### Passive measurements of fluorescence cycles

Using the method defined by Daumard et al. (2010), the fluorescence fluxes were determined from the solar spectrum reflected by the target and compared with the light reflected by a flat white horizontal board as described in the Materials and Methods section. In Fig. 8a, we observed that the fluxes at 687 and 760 nm were similar in shape but different in amplitude. Several facts should be taken into account that may explain a different behavior:

F687, peaking at almost the first peak of the fluorescence emission spectrum, reflects mainly PSII fluorescence and is fully affected by the development of NPQ under the high light conditions prevalent in Fig. 8a. One may also observe that at light intensities under 300 µmol photons m^−2^ s^−1^, that is, under relatively low light, F760 and F687 are almost superimposed (see Fig. 8c), whereas, at 1000 µmol photons m^−2^ s^−1^, F760 is about 1.5 times F687. The reabsorption of ChlF also plays an important role. Fluorescence emission recorded at 687 nm comes principally from the upper layers of the leaf, as the contribution from the deeper layers are strongly reabsorbed by chlorophyll itself due to the overlap between absorption and emission spectra. This is not the case at 760 nm where re-absorption does not occur and the recorded signal is enhanced by the contribution of the deeper layers of the leaf, as compared to 687 nm. As the effective irradiance experienced by these deeper layers is significantly reduced from its value at the very surface, they are supposed to be less affected by the development of NPQ. In fact, these two wavelengths are sampling different parts of the leaf which are excited with different light intensities.

F760 is, like F740, close to the second maximum of the fluorescence emission. This wavelength is strongly affected by the contribution of the constant PSI fluorescence emission, as shown in several works (Pfündel 1998; Agati et al. 2000; Franck et al. 2002). These authors concluded that PSI fluorescence contributes around 35–40% of F_0_. In our case, under high light conditions, the fluorescence level is also close to F_0_ (see Fig. 7a).

In addition, we should consider that the F760 excitation wavelength domain includes the spectral range over 700 nm that no one uses to excite chlorophyll fluorescence as it is impossible to excite and detect fluorescence at the same wavelength. This wavelength range is absorbed by PSI and contributes to the emission at F760, but it does not excite PSII. Thus, the contribution of PSI fluorescence at F760 is reinforced (Laisk et al. 2014; Zhen and Bugbee 2020).

### Comparison between active and passive diurnal cycles

At variance with the LIF technique, where the excitation source and experimental conditions are fixed and allow to define of a relative fluorescence yield, the SIF method retrieves fluorescence fluxes that should be divided by the radiation absorbed by the vegetation to compute a relative yield (see Eqs. 1a and 1b in Materials and Methods 5th section). However, calculating the radiation absorbed by the vegetation using remote sensing methods results in a big challenge. Nevertheless, we can get an apparent spectral fluorescence yield (ASFY) considering approximations of absorbed radiation.

**Fig. 9:**
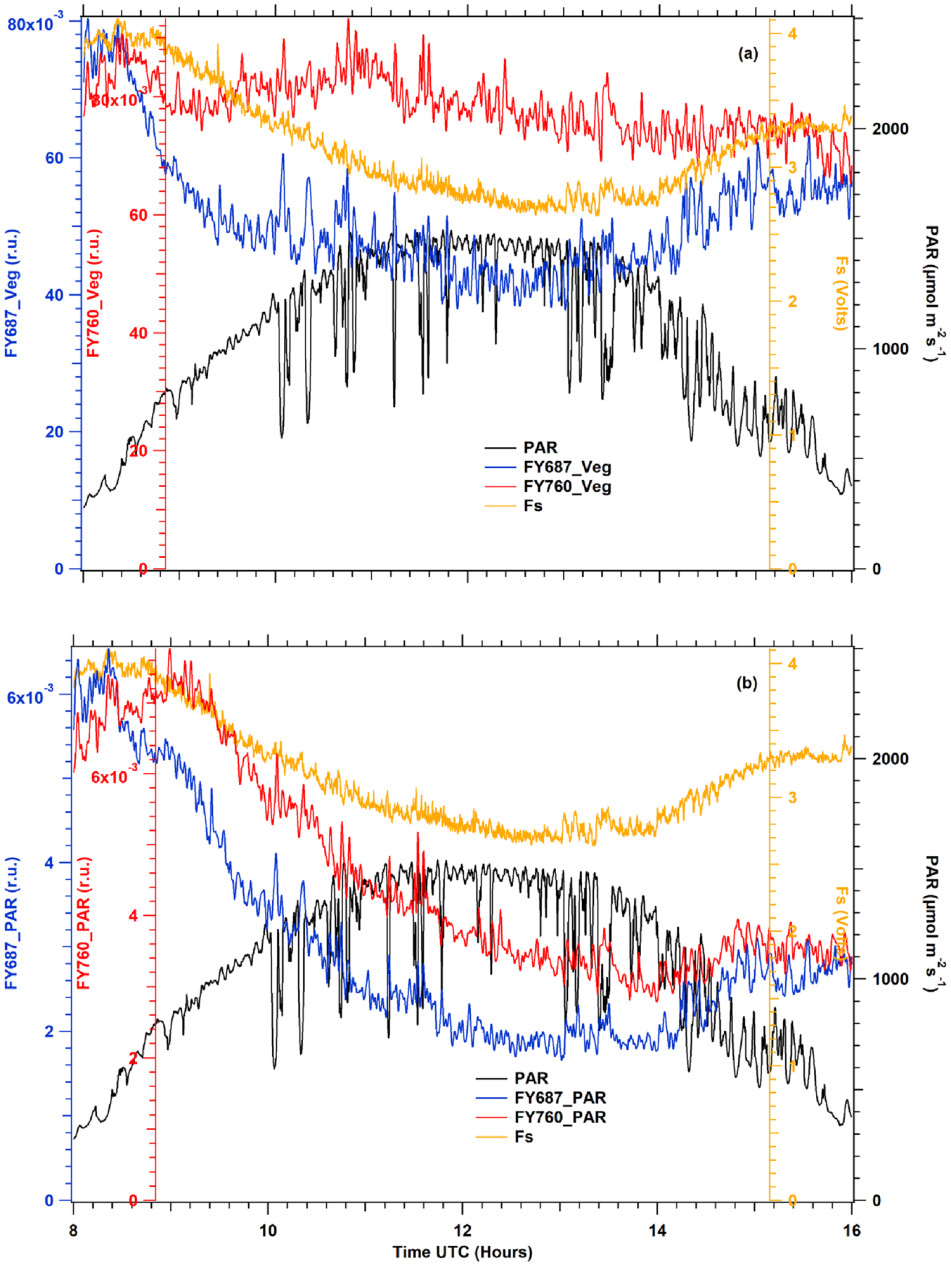
Comparisons at 56 DAP between fluorescence yields measured with the active method (orange) versus apparent fluorescence yields at 687 nm (blue) and 760 nm (red) retrieved by passive methods: (**a**) apparent fluorescence yields normalized by radiance of vegetation (**b**) apparent fluorescence yields normalized by incident PAR. Significant differences are observed between the patterns of the diurnal time courses. Similar results were obtained in all the measurements under sunny conditions

We thoroughly tested two choices that we compared in Fig. 9:

(1) Incident PAR (Fig. 9b). Regarding incident PAR, we used the radiance of the reference panel as this signal is measured by our spectrometer each time we measured the radiance of the vegetation (see Materials and Methods, 5th section). It is based on the fact that fluorescence fluxes are recovered using radiances measured over the reference panel as a proxy of the incident light, and besides that, the capture times of each measurement involved in computing passive fluorescence can be obtained using a unique sensor. Furthermore, we used the difference between the reference panel's and vegetation's radiance with almost identical results.

(2) Vegetation radiance in the 510–700 nm range as described in Materials and Methods 5th section (Fig. 9a).

We compared the fluorescence fluxes after normalization by PAR or vegetation radiance with Fs (Fig. 9). The time series of apparent spectral fluorescence yields and Fs were restricted to a time window from 8:00 to 16:00 local time to avoid artifacts in the passive data due to lower solar illumination angles. In addition, these patterns were observed on different sunny days.

Daily cycles were normalized with their respective maximum ${Fs}_{max}$ and ${FY}_{max}$. Furthermore, cubic splines were applied to calculate statistics that allow measuring the differences between Fs and the apparent yields derived from passive measurements (F.Y.s). The distances between Fs and all F.Y.s were calculated using the sum of squared residuals (SSR, see Eq. 3 below) as a criterion in model selection, which means that an SSR value closer to zero results in a better fit.

7 $$SSR=\frac{{\sum }_{{t}_{i}=8:00}^{16:00}{\left[Fs\left({t}_{i}\right)-FY\left({t}_{i}\right)\right]}^{2}}{{Fs}_{max}{FY}_{max}}$$

Time series resulting from cloudy day measurements were dropped from the analysis since no significant differences were found between the F.Y.s calculated with the incident PAR.

Considering the average of all diurnal cycles recorded, the SSR ratio between FY760_PAR and FY760_Veg is 2.62, while that between FY687_PAR and FY687_Veg is 3.13. Therefore, F.Y.s computed with vegetation radiance are significantly better than F.Y.s obtained with incident PAR (Table 2). Hence, these later ones were excluded from the following analysis.

**Table 2:** Measure of the normalized difference between apparent fluorescence yields derived from passive measurements and Fs over a diurnal cycle (Sum of squares of residuals, SSR) according to emission wavelengths (687 nm, 760 nm) and normalization method (PAR: irradiance; Veg: vegetation radiance).

DAP	FY687_PAR	FY687_Veg	FY760_PAR	FY760_Veg	FYSIF
48	677.07	409.37	112.94	157.49	133.32
51	490.76	311	380.85	258.43	234.36
52	998.98	422.32	726.5	238.95	147.8
53	797.51	232.67	364.83	23.94	51.15
54	340.74	128.98	212.99	100.65	50.53
56	1704.73	222.49	750	349.34	57.77
57	595	295.04	578.7	200.32	107.69
64	632.2	198.92	248.14	65.60	61.15
73	182.82	47.28	130	337.1	169.56
74	162.66	41.47	233.95	61.52	23.05
80	543.55	305.76	899.52	325.51	177.21
81	637.6	185.6	661.8	126.74	95.69
82	1309.19	92.78	744.26	65.84	42.15
SSR (average)	697.91	222.59	464.96	177.8	103.96

FYSIF combined the two wavelengths, 687 nm and 760 nm, normalized by vegetation radiance. Again, only sunny days of the experiment are reported

### Optimized SIF versus LIF measurements

Leaves movements due to wind may also contribute to decorrelate signals of Spectroflex (period of measurements 12 s per loop) and Ledflex (period 2 s per loop). Furthermore, Ledflex detects fluorescence emission over the whole spectral band between 650 and 800 nm (Fig. 1), which mixes the red and the far-red emissions of ChlF. To better represent the whole ChlF with the passive method, we combined the FY687 and FY760 apparent yields into a single indicator, considering their values as representative of the emission of the red and far-red ChlF bands, respectively. This approach is supported by the fact that the total emission is mainly controlled by the two independent factors related to the PSI and PSII emissions.

Ledflex transmission calculated a correction coefficient for each passive signal: 1 for FY687_Veg and 0.95 for FY760_Veg (see Materials and Methods, 2nd section, Fig. 1), and an equivalent F.Y. was calculated with the aim to more closely fit Fs time series with passive measurements. This improved F.Y. was called FYSIF and is defined as:

8 $$FYSIF=FY{687}_{Veg}*1+FY{760}_{Veg}*0.95$$

**Fig. 10:**
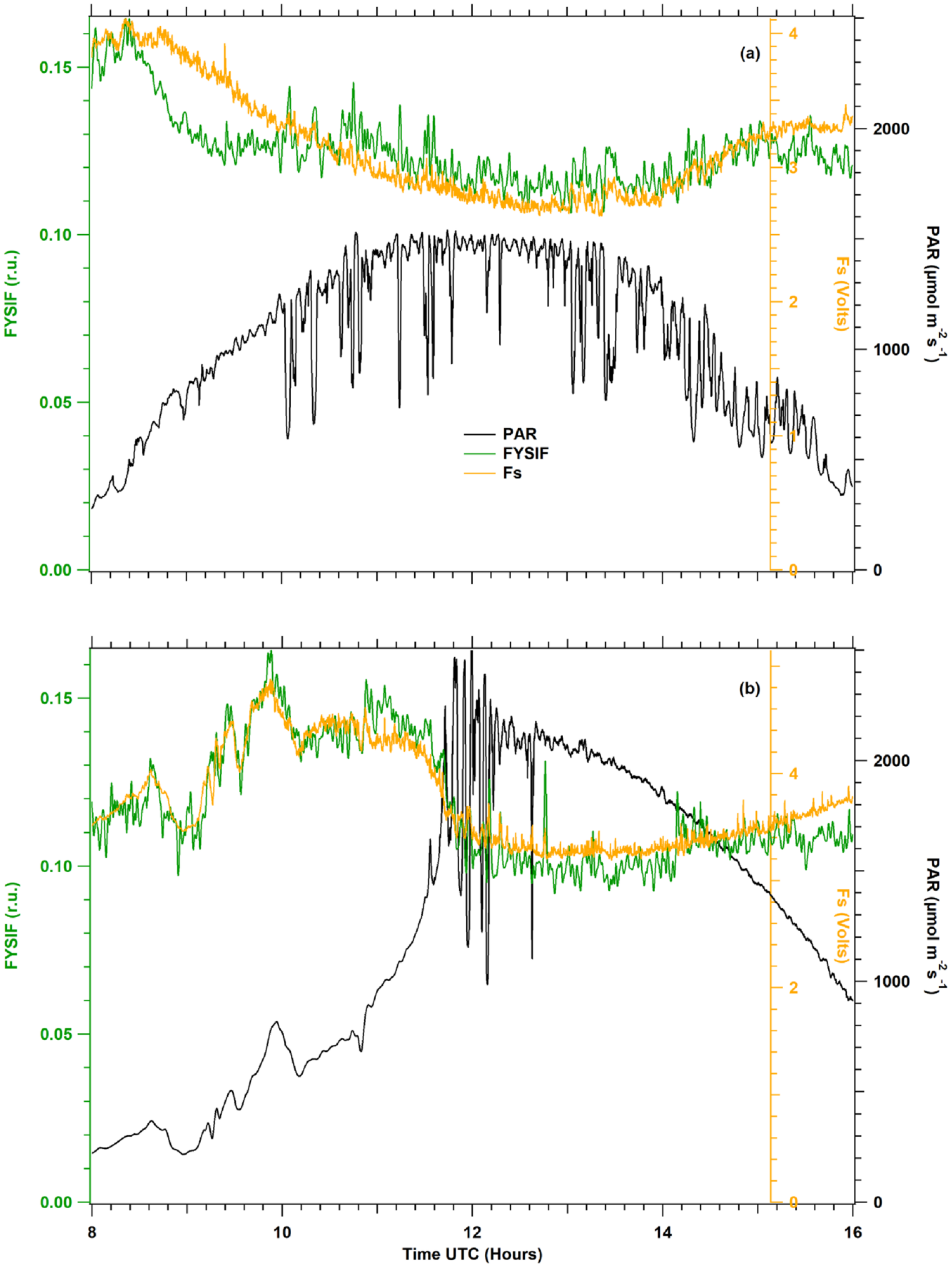
Diurnal cycles of FYSIF and Fs under sunny conditions at 56 and 64 DAP. Similar results were obtained for other days of the experiment

FYSIF time series were compared with Fs under different natural light conditions. Good agreements were found between the variables (see examples in Fig. 10). The SSR indicator was computed for all F.Y.s and sunny days of the experiment (Table 2). The last row of Table 2 shows the SSR average of each F.Y.

Except for the data corresponding to 53 DAP where the SSR computed for FY760_Veg is less than FYSIF and for 73 DAP where SSR computed for FY687_Veg is less than FYSIF, the SSR corresponding to FYSIF is less than other F.Y. models presented in Table 2.

Considering the average SSR computed for each passive fluorescence yield described in this work, the mix of passive fluorescence measurements at 687 nm and 760 nm divided by vegetation radiance (FYSIF) resulted by far in the best fit to Fs, with an SSR value of 103.96. In addition, the close results between fluorescence yields retrieved with active and passive methods implies that leaves movement seems to have only a negligible effect.

## Conclusion

The present work constitutes the first report comparing active and passive measurements of chlorophyll fluorescence by filling-in atmospheric oxygen absorption bands at 687 and 760 nm at the canopy level.

Diurnal cycles of fluorescence fluxes at 687 and 760 nm showed a similar signal-to-noise ratio but differed in magnitudes. To extract relative fluorescence yields from passive fluorescence fluxes, we used two different approaches of normalization:

- by the flux reflected by a flat and white reference surface—i.e., representative of the incident radiation,

- by the flux reflected by the vegetation.

The resulting diurnal cycles of the relative fluorescence yield at 687 nm and 760 nm significantly differed from the fluorescence yield obtained by the active method. Furthermore, dividing the fluxes by the radiance of the vegetation was found to reproduce better the actual fluorescence yield than dividing them by the radiance of the white panel. In order to better represent the spectral detection range of the active sensor, we combined the two passive fluorescence yields in the red and far-red after normalization by the vegetation radiance. This approach was successful as a good agreement was found between fluorescence yields derived from active and passive methods. Moreover, we confirmed the differences between fluorescence yields diurnal cycles at 687 nm and 760 nm that we explained by the contribution of PSI to the 760 nm emission and the reabsorption of red fluorescence, which limits the contribution of the deeper layers of the leaf at 687 nm. This also improved the confidence in the retrieval method proposed by Daumard et al. (2010), which computes fluorescence fluxes using only four channels to retrieve fluorescence at 687 nm and three channels for fluorescence at 760 nm. Additionally, the low computational cost allows to retrieve fluorescence in real time.

## Data Availability

The data that support the findings of this study are openly available in Dataverse CGIAR repository at: Diurnal cycles of passive and active fluorescence measurements at canopy level in potato crops. https://doi.org/10.21223/5RL85W

## Abbreviations

SIF: Solar-induced fluorescence
LIF: Laser or LED-induced fluorescence
ASFY: Apparent spectral fluorescence yield
ChlF: Chlorophyll fluorescence
PAR: Photosynthetically active radiation
Fs: Stationary fluorescence yield
F760: Fluorescence fluxes at 760 nm
FY760: Fluorescence Yield at 760 nm
ASFY760: Apparent spectral fluorescence yield at 760 nm
F687: Fluorescence fluxes at 687 nm
FY687: Fluorescence Yield at 687 nm
ASFY687: Apparent spectral fluorescence yield at 687 nm
IDE: Integrated development environment
DAQ: Data acquisition
SNR: Signal to noise ratio
SSR: Sum of squared residuals
O_2_A: Oxygen A absorption band
O_2_B: Oxygen B absorption band
PSI: Photosystem I
PSII: Photosystem II
NQP: Non-photochemical quenching
